# Broad-spectrum resistance mechanism of serine protease Sp1 in *Bacillus licheniformis* W10 *via* dual comparative transcriptome analysis

**DOI:** 10.3389/fmicb.2022.974473

**Published:** 2022-10-04

**Authors:** Lina Yang, Chun Yan, Shuai Peng, Lili Chen, Junjie Guo, Yihe Lu, Lianwei Li, Zhaolin Ji

**Affiliations:** ^1^College of Horticulture and Plant Protection, Yangzhou University, Yangzhou, China; ^2^The Key Laboratory of Biotechnology for Medicinal Plants of Jiangsu Province, School of Life Sciences, Jiangsu Normal University, Xuzhou, China

**Keywords:** *Bacillus licheniformis* W10, Sp1 protein, broad-spectrum resistance mechanism, transcriptome analysis, disease resistance

## Abstract

Antagonistic microorganisms are considered to be the most promising biological controls for plant disease. However, they are still not as popular as chemical pesticides due to complex environmental factors in the field. It is urgent to exploit their potential genetic characteristics and excellent properties to develop biopesticides with antimicrobial substances as the main components. Here, the serine protease Sp1 isolated from the *Bacillus licheniformis* W10 strain was confirmed to have a broad antifungal and antibacterial spectrum. Sp1 treatment significantly inhibited fungal vegetative growth and damaged the structure of hyphae, in accordance with that caused by W10 strain. Furthermore, Sp1 could activate the systemic resistance of peach twigs, fruits and tobacco. Dual comparative transcriptome analysis uncovered how Sp1 resisted the plant pathogenic fungus *Phomopsis amygdali* and the potential molecular resistance mechanisms of tobacco. In PSp1 vs. *P. amygdali*, RNA-seq identified 150 differentially expressed genes (DEGs) that were upregulated and 209 DEGs that were downregulated. Further analysis found that Sp1 might act on the energy supply and cell wall structure to inhibit the development of *P. amygdali*. In TSp1 vs. Xanthi tobacco, RNA-seq identified that 5937 DEGs were upregulated and 2929 DEGs were downregulated. DEGs were enriched in the metabolic biosynthesis pathways of secondary metabolites, plant hormone signal transduction, plant–pathogen interactions, and MAPK signaling pathway–plant and further found that the genes of salicylic acid (SA) and jasmonic acid (JA) signaling pathways were highly expressed and the contents of SA and JA increased significantly, suggesting that systemic resistance induced by Sp1 shares features of SAR and ISR. In addition, Sp1 might induce the plant defense responses of tobacco. This study provides insights into the broad-spectrum resistance molecular mechanism of Sp1, which could be used as a potential biocontrol product.

## Introduction

The United Nations Food and Agriculture Organization estimates that the global economic damages due to plant diseases are more than 22 billion dollars and that reduced agricultural production is exacerbating global hunger and endangering livelihoods. Chemical pesticides are widely used in disease prevention and control due to their quick effects. However, long-term, unreasonable application could easily produce pesticide residue, pathogen resistance, and adverse effects on human and animal health and the environment. Therefore, biological control is one of the most promising methods to control plant diseases from a security perspective (Aktuganov et al., 2014; Syed Ab Rahman et al., 2018; He et al., 2021). *Bacillus* spp. widely distributed in various ecological environments, such as plants and soil, and could colonize plant environments, inhibit pathogen infection, induce plant resistance, be compatible with chemical pesticides and produce endospores with strong stress resistance. *Bacillus* spp. are widely used biocontrol bacteria, among which *B. licheniformis* is a member (Fira et al., 2018; Won et al., 2021). There have been many reports on the biocontrol mechanism of *B. licheniformis*, including antagonism, competition, lysozyme, induced disease resistance, and growth promotion (Pérez-García et al., 2011; Muslim et al., 2016; Jeong et al., 2017; Won et al., 2019; Ji et al., 2020a). Studies have confirmed that *B. licheniformis* has certain control effects on plant diseases caused by fungi, oomycetes, bacteria, viruses and nematodes (Asraful Islam et al., 2010; Slimene et al., 2015; Bhai et al., 2019; Abdelkhalek et al., 2020; Panichikkal et al., 2021; Upadhyay and Mohan, 2021).

Biocontrol strains have excellent effects on research conditions, and various biopesticides have been developed. However, they were still not as popular as chemical pesticides in a survey covering nearly two decades. Complex environmental factors in the field easily affect their development and metabolism, such as temperature, light, humidity, nutrients, chemical substances, and various stress conditions (Someya, 2008). Therefore, it is urgent to explore their potential genetic characteristics and excellent properties, and developing biopesticides with antimicrobe substances as the main components, transferring these substances into plants to obtain resistant transgenic plants, or constructing efficient biocontrol engineering bacteria are important directions for the biological control of diseases.

The most prominent feature of *Bacillus* is their abundant metabolic system and ability to produce various extracellular proteins, including chitinase, glucanase, peptide, lipopeptide and other types of antifungal proteins (Leelasuphakul et al., 2006; Gomaa, 2012; Fira et al., 2018). Chitinase and glucanase were reported to be major mucolytic enzymes that dissolve fungal cell walls (Ji et al., 2020b). Lipopeptides such as iturins and surfactin from *Bacillus* are widely known for targeting the host cell wall that causes its collapse and the membrane through pore formation, ultimately leading to cell death and inhibiting fungal growth (Someya, 2008; Tian et al., 2021). Wang et al. (2014) identified a 55 kDa antifungal protein produced by the *B. licheniformis* HS10 strain that was a carboxypeptidase and had significant inhibitory effects on eight different plant pathogenic fungi. The 31 kDa antifungal protein with hydrolyzing activity on casein in *B. licheniformis* BS-3 inhibited the growth of *Aspergillus niger*, *Magnaporthe oryzae*, and *Rhizoctonia solani* (Cui et al., 2012). The 48 kDa serine protease MG-3A purified from the *B. amyloliquefaciens* MG-3 strain exhibited promising antifungal activity against four kinds of fungi and effectively extended the shelf life of loquat fruit (Yan et al., 2021). *B. licheniformis* W10 strains were isolated from the tomato rhizosphere by our lab and have significant inhibitory effects on *Botrytis cinerea*, *Monilinia fructicola*, and *Sclerotinia sclerotiorum*. Studies have shown that treatment with W10 can cause malformed mycelium, extravasation of protoplasm, and changes in cell membrane permeability, and can inhibit vegetative growth and conidial germination (Ji et al., 2015, 2020a). Furthermore, an approximately 48 kDa extracellular antifungal protein that encoded serine proteases (named the Sp1 protein) was purified from *B. licheniformis* W10 strain, and it was found that Sp1 had a strong inhibitory effect on *B. cinerea*, good thermal stability and pH tolerance, demonstrating that the Sp1 protein could be a potential biocontrol enzyme of plant pathogenic fungi (Ji et al., 2020b). However, the underlying molecular regulatory mechanism of the antifungal protein of *Bacillus* is still not clear.

Serine proteases depend on their serine residue for catalytic activity are widespread and numerous. Most of them were grouped into about six clans due to protein three-dimensional structures and the order of catalytic residues in the polypeptide chain, including SA, SB (subtilisin), SC, SE, SF, and SG family (Rawlings and Barrett, 1994). W10-Sp1 protein had a conserved S8 domain which belongs to subtilisin family. The subtilisin family that first observed in *B. subtilis* is the second largest family of serine proteases, present in many organisms, however, mostly found in plant, such as in tomato, potato, *Arabidopsis*, grape, soybean and tobacco (Figueiredo et al., 2018). Plant subtilases play important roles in its development functions, including the growth of seeds and fruits and cell wall modification, besides that, they have got more attention in the plant response to the biotic and abiotic stress (Schaller et al., 2012). Studies have been reported that they mainly response to drought and salt stress (Liu and Howell, 2010; Budič et al., 2013). Accumulation of the subtilases were found after infection with the pathogens in tomato, grapevine and cotton (Vera et al., 1989; Monteiro et al., 2013; Duan et al., 2016), and further some substrates were identified such as systemin and the leucine-rich repeat (LRP), initiated defense signaling pathway and activated the expression of defense-related genes (Bergey et al., 1996; Tornero et al., 1996; Ryan, 2000). In addition, studies have showed that some specific subtilases are involved in plant programmed cell death (PCD) (Fernández et al., 2015). Besides that, subtilases are also found in pathogens as one of the virulence factors to disrupt host cell membrane during infection process, or degraded host PR protein, or as an avirulent to trigger a strong plant defense reaction (Olivieri et al., 1998; Walter et al., 2010; Chalfoun et al., 2013). Above all, subtilases perform multiple functions whether in plants or in pathogens. However, the specific mechanisms in biocontrol microorganism need to be further studied.

In this study, we found that W10-Sp1 had a broad antifungal effect, further inhibiting the growth of some bacteria and activating the systemic resistance of peach twigs, fruits and tobacco. Then, the broad-spectrum resistance molecular mechanism of the Sp1 protein was explored through dual comparative transcriptome analysis.

## Materials and methods

### Strains, culture conditions and purification of the W10-Sp1 protein

Strains of bacteria, including *Bacillus licheniformis* W10 (CGMCC No. 14859), *Xanthomonas arboricola* pv. *pruni*, *Pseudomonas cichorii*, *Pseudomonas syringae* pv. *tomato*, *Acidovorax avenae* subsp. *citrulli* and *Pectobacterium carotovorum* subsp. *carotovorum*, and fungi, including *Botrytis cinerea, Phomopsis amygdali*, *Rhizoctonia solani*, *Monilinia fructicola*, *Sclerotina sclerotiorum*, *Glomerella cingulata*, *Rhizopus stolonifer*, *Fusarium moniliforme*, *Alternaria cerasi* and *Gaeumannomyces graminis*, were preserved at the Peach Disease Research Laboratory, College of Horticulture and Plant Protection, Yangzhou University, China. Fungal strains were cultured at 25°C in potato dextrose agar (PDA) medium. Bacterial strains were cultured at 28°C in nutrient agar (NA) medium. The W10-Sp1 protein was purified according to our previous work (Supplementary Figure S1; Ji et al., 2020b). Peach [*Prunus persica* (L.) Batsch] (“Liutiaobaifeng”) twigs were used within 1- to 2-year old in this study. Peach fruits were from peach experimental and demonstration bases at Yangshan, Wuxi, China. Xanthi tobacco (*Nicotiana tabacum* L. “Xanthi”) plants were potted at 25°C with 80% relative humidity for 6–8 leaf stage.

### Antagonism assay of the W10-Sp1 protein against different fungal/bacterial strains

In confrontation assays against fungus, the different plant pathogenic fungal blocks (6 mm) were separately inoculated on the center of PDA plates (diameter was 9 cm). Then, the sterilized paper disks were symmetrically placed on both sides of the fungal blocks, one side was dropped with 10 μl of sterile water, and the other side was placed in 10 μl of purified W10-Sp1 protein solution at a concentration of 20 mg/L. The plates were cultured at 25°C until the vegetative mycelium overgrew, and the inhibitory diameters were measured.

In confrontation assays against bacteria, overnight cultures of plant pathogenic bacteria (OD_600_ = 0.8) were mixed with NA agar medium and poured into plates with a diameter of 9 cm. Then, the sterilized paper disks were symmetrically placed on both sides of the center, one side was dropped with 10 μl of sterile water, and the other side was placed in 10 μl of purified W10-Sp1 protein solution at a concentration of 20 mg/L. After culturing at 28°C for 24 h, the inhibitory diameters were measured. All treatments were replicated three times.

### EC_50_ measurement

W10-Sp1 protein was separately added to PDA medium at concentrations of 25, 50, 100, 250, and 500 μg/ml, and sterile water was used as a control. Then, the hyphal blocks were inoculated in the center of the plates and cultured at 25°C in the dark until the control strains overgrew on the PDA medium. When the EC_50_ of Sp1 protein to *G. graminis* was measured, the concentrations of W10-Sp1 protein were 0.25, 0.5, 1.0, 5.0, and 25 mg/L. The inhibitory rate (%) = [(control growth diameter-treated growth diameter)/(control growth diameter − 6)] × 100%. The concentrations of W10-Sp1 protein were logarithmically transformed to 10 as the abscissa, and the probability value corresponding to the inhibitory rate was used as the ordinate to obtain the regression equation. When the inhibitory rate was 50%, the corresponding concentration value was the EC_50_ value.

### RNA extraction and RNA sequencing

To explore the mechanism of how the W10-Sp1 protein resisted the plant pathogenic fungus, *P. amygdali* was used as an example in this study. Hyphal blocks of *P. amygdali* were cultured in liquid PDA medium at 25°C for 36 h, and then W10-Sp1 protein solution was added at a concentration of 100 mg/L for another 36 h (PSp1). Equal amounts of sterile water were used as a control (PCK). To explore the systemic resistance mechanism of the W10-Sp1 protein, W10-Sp1 protein at a concentration of 100 mg/L was injected into the lower leaf of Xanthi tobacco, and sterile water was injected into the control. Then, the upper leaf was used for further research. Three biological replicates were performed, and RNA was extracted from 12 samples with an RNA kit (Cat no. 12183018A, Invitrogen, United States). The DNA library was constructed and sequenced. The adapter reads were removed from the raw sequencing data, along with the reads that could not be used to determine the base information and low-quality reads; thus, clean reads were obtained. The clean reads were aligned to the reference genomes of *P. amygdali* and Xanthi tobacco using HISAT2 software. The FPKM values were used to estimate the sequencing depth and gene length.

### Differentially expressed genes analysis

Differentially expressed genes (DEGs) in treated samples vs. CK were determined by DESeq2 software. GO (Gene Ontology) terms and KEGG (Kyoto Encyclopedia of Genes and Genomes) pathway enrichment of DEGs were analyzed by clusterProfile, and the PPI (protein–protein interaction) networks of DEGs were predicted according to STRING software.

### Quantitative reverse transcription PCR analysis

Differentially expressed genes were randomly selected for reverse transcription PCR (qRT-PCR) to verify the accuracy of the dual transcriptome data. RNA was extracted from the same samples and reverse transcribed into cDNA as the templates for qRT-PCR. qRT-PCR was performed on a Bio-Rad CFX96 instrument (Bio-Rad, United States) according to the manufacturer’s instructions. All experiments were performed for three independent replicates. The primers used are listed in Supplementary Table S5.

### Conductivity measurement

The same amount of hyphal blocks were put into 40 ml of liquid PDA medium and cultured at 25°C and 180 r/min for 48 h. The plates were filtrated and washed with sterile water three times to remove the medium. Then, the hyphal blocks were placed into 5 ml of W10-Sp1 protein solution with EC_50_ or 2EC_50_ concentrations and cultured at 25°C and 180 r/min, and the conductivity was detected after 2, 4, 6, 12, and 24 h of culturing within a DDS-307 conductivity meter. Finally, the samples were boiled for 0.5 h, and the conductivity was detected after cooling. Relative conductivity = (conductivity before water bath/conductivity after water bath) × 100%. All treatments were replicated three times.

### Salicylic acid and jasmonic acid content measurement with high-performance liquid chromatography–tandem mass spectrometry method

Xanthi tobacco at the 8–10 leaf stage was selected for injection with 50 μl of W10-Sp1 at a concentration of 100 mg/L or the same amount of sterile water. The upper leaves were taken at 12 hpi and used for the extraction of plant hormones and content measurement which were referred to the reference (Pan et al., 2010). Samples were quantified by high-performance liquid chromatography–tandem mass spectrometry (HPLC-MS/MS) analysis using Poroshell 120 SB-C18 (2.1 × 150, 2.7 μm). The gradient elution program: 20% A (0.1% formic acid-methyl alcohol) at 0∼1 min; 20% A∼80% A at 1∼9 min; 80% A at 9∼10 min; 80% A∼20% A at 10∼10.1 min; 20% A at 10.1∼15 min. The peak flow of the standard of SA was at ∼5.61 min; of the standard of JA was at ∼6.22 min.

## Results

### Inhibitory effects of purified W10-Sp1 protein on 10 kinds of plant pathogenic fungi

**FIGURE 1:**
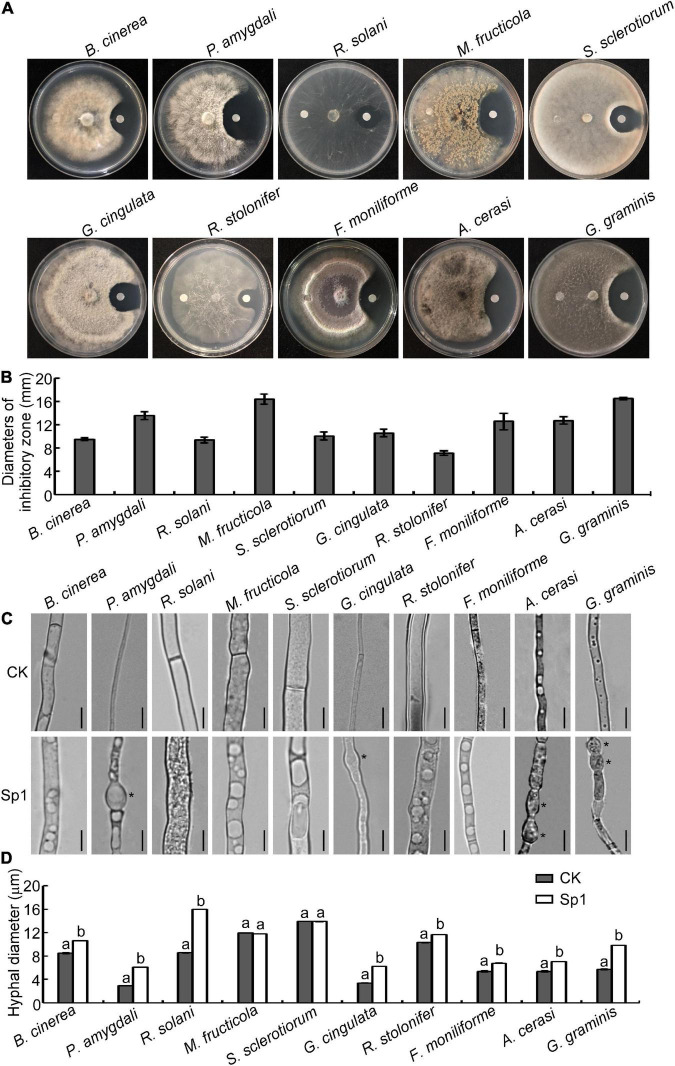
W10-Sp1 protein significantly affected the mycelial growth and the hyphal morphology of 10 kinds of fungi. **(A)** Hyphal cakes with diameters of 6 mm of 10 kinds of fungi were inoculated in the center of PDA plates. Sterilized paper disks were placed equidistant on both sides of the hyphal block. The left side was treated with sterile water, and the right side was treated with purified W10-Sp1 protein. Photographs were taken to observe the inhibition effect of W10-Sp1 after fungi overgrew the plate. **(B)** Measurement of the diameters of the inhibitory zone. Experiments were repeated three times with similar results. **(C)** Hyphal blocks of 10 kinds of fungi were separately placed into liquid PDA medium and shaken at 25°C and 160 rpm for 48 h. Then, they were treated with the purified W10-Sp1 protein in PDA medium and shaken under the same conditions for another 24 h. Sterile water was used as a CK (control). The morphology of single hyphae was separately observed under the microscope at 40×. Asterisks indicate malformed positions. Bar = 20 μm. **(D)** Measurement of hyphal diameter with different treatments under the microscope. Letters represent significant differences. Experiments were repeated three times with similar results (Duncan’s new multiple range test, *p* < 0.05).

Our previous work demonstrated that strains of *B. licheniformis* W10 and its extracellular antifungal crude protein had a better biocontrol effect to inhibit gray mold caused by *B. cinerea* and peach brown rot caused by *M. fructicola* (Ji et al., 2015, 2020a) and purified a protein, Sp1, which encoded a serine protease from the extracellular crude protein and significantly inhibited the mycelial growth of *B. cinerea* (Ji et al., 2020b). Therefore, we wondered whether the Sp1 protein could be a broad-spectrum antifungal protein. Purified W10-Sp1 protein was used to explore its effects on 10 kinds of different plant pathogenic fungi, including *B. cinerea, P. amygdali*, *R. solani*, *M. fructicola*, *S. sclerotiorum*, *G. cingulata*, *R. stolonifer*, *F. moniliforme*, *A. cerasi* and *G. graminis*. The inhibitory effects of W10-Sp1 on the vegetative mycelia of these fungi were observed by confrontation culture on PDA medium at 25°C. The results showed that W10-Sp1 had the most obvious inhibitory effects on *G. graminis* and *M. fructicola*, and the diameters of the inhibitory zones were 16.5 mm and 16.4 mm, respectively. This was followed by *P. amygdali* (13.6 mm), *A. cerasi* (12.7 mm), *F. moniliforme* (12.6 mm), *G. cingulata* (10.6 mm), *S. sclerotiorum* (10.0 mm), *B. cinerea* (9.5 mm), *R. solani* (9.4 mm), and *R. stolonifer* (7.2 mm) (Figures 1A,B).

Then, the hyphal blocks of these fungi were separately cultured in liquid PDA medium for 48 h at 25°C and then treated with W10-Sp1 protein or sterile water for another 24 h, and the single hyphal morphology was observed under a microscope. The results showed that the W10-Sp1 protein affected hyphal morphology in three ways: some were malformed and swollen in *P. amygdali*, *G. cingulata*, *A. cerasi* and *G. graminis* (Figure 1C); hyphal diameter increased in *B. cinerea, P. amygdali*, *R. solani*, *G. cingulata*, *R. stolonifer*, *F. moniliforme*, *A. cerasi*, and *G. graminis* (Figures 1C,D); and large or more vesicle-like structures occurred in the hyphae in nearly all 10 kinds of fungi (Figure 1C).

In addition, a certain amount of spore suspension was treated with W10-Sp1 protein at a final concentration of 50 mg/L, and the germination rate was observed at 12 hours post-inoculation (hpi) in hollow glasses, respectively. The results found that W10-Sp1 significantly inhibited the spore germination of those fungi compared with that of the untreated fungi; the inhibitory rates were between 58.6∼72.9%, and the highest was that of *M. fructicola* (Table 1). Overall, the results indicated that W10-Sp1 has a stronger inhibitory effect on these 10 kinds of plant pathogenic fungi.

**TABLE 1:** Effects of purified W10-Sp1 protein on spore germination rates of different plant pathogenic fungi.

Strains	Spore germination rates (%)^†^		Inhibitory rates (%)^‡^
	CK	W10-Sp1	
*B. cinerea*	59.7 ± 3.5^a^	24.7 ± 2.9^b^	58.6 ± 5.0
*P. amygdali*	52.7 ± 4.8^a^	18.8 ± 1.8^b^	64.3 ± 3.4
*M. fructicola*	50.7 ± 2.9^a^	13.7 ± 3.1^b^	72.9 ± 6.0
*G. cingulata*	54.3 ± 3.8^a^	17.3 ± 2.4^b^	68.1 ± 4.4
*R. stolonifer*	62.0 ± 4.6^a^	22.0 ± 3.2^b^	64.5 ± 5.1

^†^Spore suspensions with the concentration of 1.0 × 10^5^ spores/ml of different fungi were treated with sterile water (CK) or W10-Sp1 protein with a final concentration of 50 mg/L, separately. Inoculated 40 μl on the concavity slides, and spore germination rates were observed at 12 hours post-inoculation (hpi). Germination rates = (number of spore germination observed/number of total spores observed) × 100%. Lowercase represents significant difference. Experiments were repeated three times with similar results. Duncan’s new multiple range test, p < 0.05.

^‡^Inhibitory rates = [(spore germination rates of control − spore germination rates of treatment with Sp1 protein)/spore germination rates of control] × 100%.

### The W10-Sp1 protein had significant differences in EC_50_ value among different plant pathogenic fungi

The antifungal effects of the W10-Sp1 protein on these 10 kinds of plant pathogenic fungi became much stronger with increasing concentrations. However, there were certain differences in inhibitory effects among the different fungi (Supplementary Figure S2). EC_50_ represents concentrations required for a half maximal effect. Linear regression analysis and EC_50_ values were calculated in different fungi treated with Sp1 protein at different concentrations. The results showed that the W10-Sp1 protein had the best anti-*G. graminis* effect, with an EC_50_ value of 4.2 mg/L, followed by *P. amygdali* (82.8 mg/L), *G. cingulata* (106.6 mg/L), *A. cerasi* (133.8 mg/L), *R. solani* (183.8 mg/L), *M. fructicola* (185.6 mg/L), *R. stolonifer* (204.9 mg/L), *S. sclerotiorum* (244.9 mg/L), *F. moniliforme* (298.8 mg/L) and *B. cinerea* (398.2 mg/L) (Table 2). These results indicated that the W10-Sp1 protein could be used as a broad-spectrum antifungal drug in the future.

**TABLE 2:** EC_50_ values of purified W10-Sp1 against different plant pathogenic fungi.

Strains	Linear regression equation	*R* ^2^	EC_50_
*B. cinerea*	*y* = 0.8943 + 1.5791*x*	0.9560	398.2
*P. amygdali*	*y* = −0.3619 + 2.7956*x*	0.9941	82.8
*R. solani*	*y* = 0.6318 + 1.9291*x*	0.9976	183.8
*M. fructicola*	*y* = 3.0350 + 0.8662*x*	0.9955	185.6
*S. sclerotiorum*	*y* = 1.8022 + 1.3928*x*	0.9303	244.9
*G. cingulata*	*y* = 0.0607 + 2.4360*x*	0.9570	106.6
*R. stolonifer*	*y* = 1.9864 + 1.3038*x*	0.9994	204.9
*F. moniliforme*	*y* = 0.9520 + 1.6353*x*	0.9072	298.8
*A. cerasi*	*y* = 3.6248 + 0.6467*x*	0.9908	133.8
*G. graminis*	*y* = 4.1025 + 1.4408*x*	0.9242	4.2

Logarithm of 10 of concentrations of W10-Sp1 protein was used as abscissa and probability values corresponding to inhibitory rate were used as ordinate to obtain regression equation. When inhibitory rate was 50%, the corresponding concentration value was EC_50_ value.

### Inhibitory effects of W10-Sp1 protein on four kinds of plant pathogenic bacteria

**FIGURE 2:**
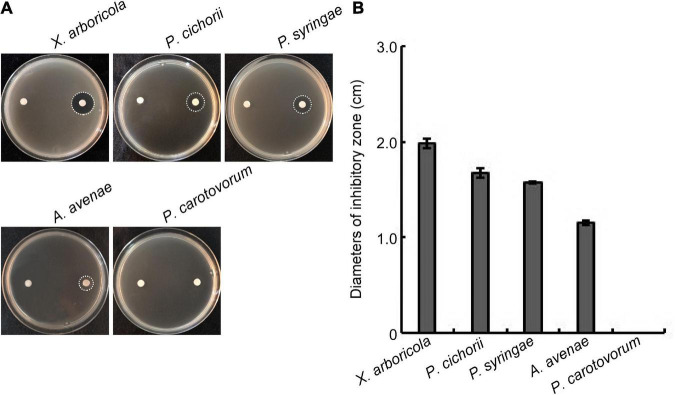
Effects of the W10-Sp1 protein on different plant pathogenic bacteria. **(A)** Bacterial suspensions (OD_600_ = 0.8) of different strains were mixed with NA medium. Two disinfected paper disks were separately placed on both sides of the plates. Sterilized water was used to treat the left sides; W10-Sp1 protein was used to treat the right sides. Photographs were taken at 2 days post-inoculation (dpi). **(B)** Measurement of the diameters of the inhibitory zone. Experiments were repeated three times with similar results.

We were then determined whether the W10-Sp1 protein had an inhibitory effect on plant pathogenic bacteria. Strains of *X. arboricola* pv. *pruni*, *P. cichorii*, *P. syringae* pv. *tomato*, *A. avenae* subsp. *citrulli* and *P. carotovorum* subsp. *carotovorum* were mixed with NA medium with treated paper disks with or without W10-Sp1 solution (20 mg/L) and cultured at 28°C for 48 h. The W10-Sp1 protein had significant inhibitory effects on *X. arboricola* (19.8 mm), *P. cichorii* (16.7 mm), *P. syringae* (15.7 mm), and *A. avenae* (12.7 mm), but had no effect on *P. carotovorum* (Figures 2A,B). These results suggested that W10-Sp1 also had- antibacterial effects.

### Transcriptome analysis of W10-Sp1 protein anti-fungal mechanism

**FIGURE 3:**
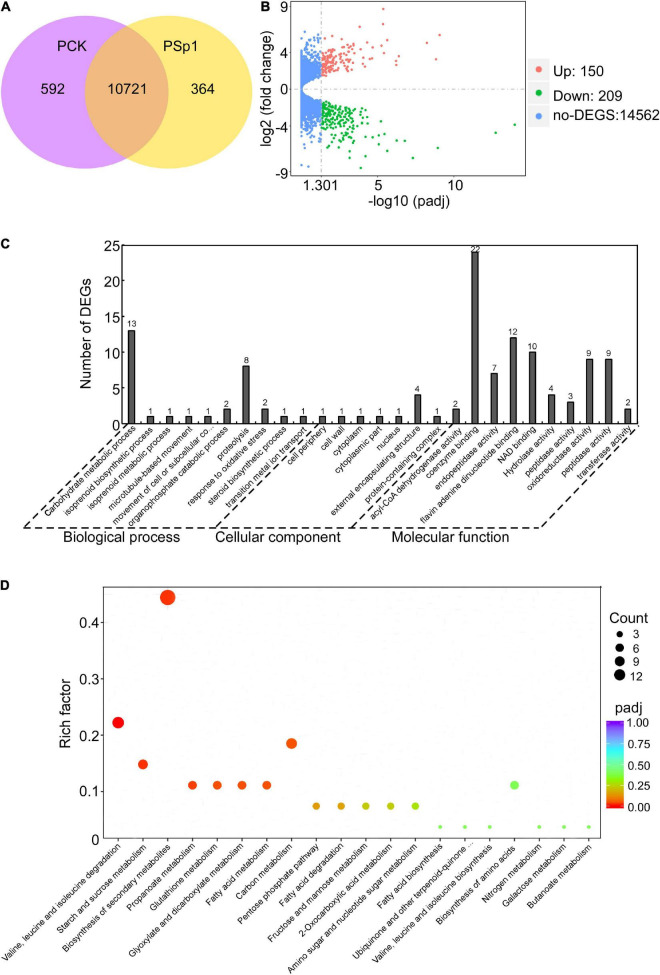
Transcriptome analysis of W10-Sp1 treating the *P. amygdali* ZN32 strain (PSp1) vs. the ZN32 strain (PCK). **(A)** Venn diagram presenting the overlap of expressed genes between PCK and PSp1. Purple represents the number of expressed genes of PCK; yellow represents the number of expressed genes of PSp1; orange represents the overlapping expressed genes of PSp1 and PCK. **(B)** Volcano plot of DEGs between PSp1 and PCK. Splashes represent different genes, where red shows significantly upregulated genes after purified Sp1 protein was used to treat hyphae of *P. amygdali* ZN32, green shows downregulated genes, and blue shows no significant difference between the samples of PSp1 and PCK. **(C)** GO analysis of DEGs. The X-axis represents the analysis categories, including biological process, molecular function and cellular component. The *Y*-axis represents the number of DEGs. **(D)** Bubble diagram of the KEGG pathway analysis of DEGs. The *X*-axis represents the top 20 KEGG signaling pathways. The *Y*-axis represents enriched factors. The closer the color was to red, the smaller the padj value and the higher the significance level.

Based on the better inhibitory effect of the W10-Sp1 protein on *P. amygdali*, it was selected in this study to further explore its anti-fungal mechanism. Hyphal blocks of *P. amygdali* were cultured in liquid PDA medium for 48 h, and then 100 mg/L W10-Sp1 protein solution was added and shaken at 25°C for another 24 h (PSp1). Medium without W10-Sp1 protein was used as a control (PCK). RNA was extracted to analyze the differentially expressed genes (DEGs) in the whole genome of *P. amygdali*, which was obtained by our lab (in preparation). The average total numbers of clean reads were 45.38 Mb and 42.48 Mb; the Q20 values were 99.02% and 99.04%; the Q30 values were 97.86% and 97.89%; and the total mapping ratios were 97.94% and 98.07% in the three PCK (PCK-1, PCK-2, PCK-3) samples and the three PSp1 (PSp1-1, PSp1-2, PSp1-3) samples, respectively (Supplementary Table S1). Eight genes were selected at random to verify the accuracy of RNA-seq by qRT-PCR under similar treatments. The qRT-PCR results showed that the expression levels of GME1740_g, GME8443_g, and GME8738_g were upregulated, and the expression levels of the other five genes were downregulated, which was consistent with the RNA-seq results (Supplementary Figure S3), indicating that the RNA-seq data were reliable. A total of 11,677 transcripts were obtained, among which 10,721 were present in both conditions. However, 364 and 592 transcripts were specifically expressed in PSp1 and PCK samples, respectively (Figure 3A). DEG analysis found that 150 transcripts were upregulated, 209 transcripts were downregulated, and most genes (14562 transcripts) had no differential expression (| log2 fold change (FC)| > 0, padj < 0.05) (Figure 3B). Furthermore, GO and KEGG pathway enrichments were analyzed. The top 10 GO enrichments were coenzyme binding, carbohydrate metabolic process, flavin adenine dinucleotide binding, NAD binding, oxidoreductase activity, peptidase activity, proteolysis, endopeptidase activity, external encapsulating structure and hydrolase activity (Figure 3C). The top 10 KEGG enrichments were biosynthesis of secondary metabolites, valine, leucine and isoleucine degradation, carbon metabolism, starch and sucrose metabolism, propanoate metabolism, glutathione metabolism, glyoxylate and dicarboxylate metabolism, fatty acid metabolism, biosynthesis of amino acids and pentose phosphate pathway (Figure 3D).

### W10-Sp1 treatment might affect the energy supply and the cell wall structure of *Phomopsis amygdali*

**FIGURE 4:**
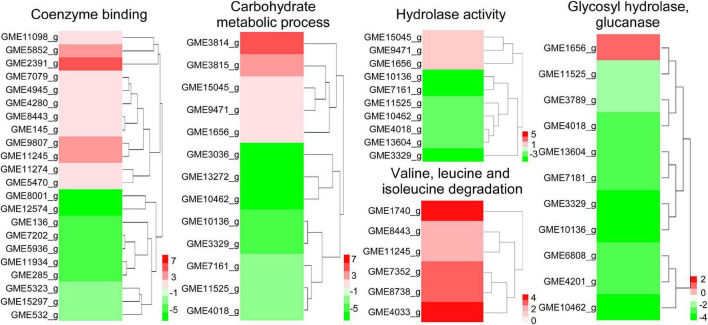
Heatmap analysis of DEGs related to key enzymes of the TCA cycle, glycosyl hydrolase and glucanase. The red color represents DEGs with upregulation, and the green color represents DEGs with downregulation.

Differentially expressed genes in coenzyme binding included 12 upregulated and 10 downregulated genes under W10-Sp1 treatment of the hyphae of *P. amygdali* compared with untreated hyphae. In carbohydrate metabolic processes, five DEGs were upregulated, and eight DEGs were downregulated. There were three upregulated and seven downregulated DEGs in hydrolase activity and six DEGs in valine, leucine and isoleucine degradation processes that were all upregulated (Figure 4). In addition, we found that genes expressing glycosyl hydrolase (an important energy production enzyme) and glucanase (cell wall glucan hydrolase) were notably downregulated (Figure 4). These results indicated that W10-Sp1 treatment might affect the energy supply and the cell wall structure of *P. amygdali*.

### The W10-Sp1 protein caused cell damage in nine kinds of plant pathogenic fungi

**FIGURE 5:**
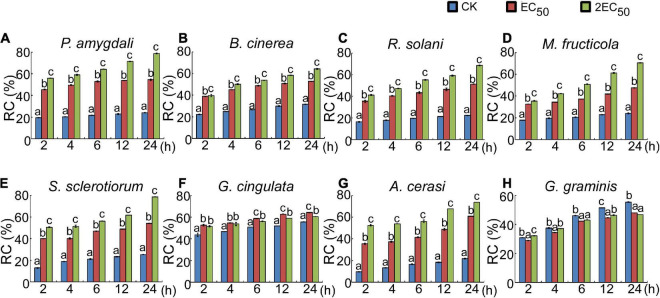
Effects of the W10-Sp1 protein on cell damage of different plant pathogenic fungi. The hyphal blocks of each fungal strain were shaken in liquid PDA medium at 25°C and 160 r/min for 48 h and washed repeatedly with sterile water, and the surface medium of mycelium pellets was removed. Then, they were placed in purified W10-Sp1 solution at EC_50_ and 2EC_50_ concentration, and the conductivity of the solution was measured after coculture for 2, 4, 6, 12, and 24 h. Finally, the cocultures were heated in boiling water for 30 min, and the final conductivity was measured after cooling **(A–H)**. Relative conductivity = (conductivity before the water bath/conductivity after the water bath) × 100%. Experiments were repeated three times with similar results (Duncan’s new multiple range test, *p* < 0.05).

The above studies showed that the W10-Sp1 protein significantly affected the hyphal morphology of the fungi and DEGs of cell wall related-genes which might be due to the damage of cell structure. Ten different kinds of fungi were cultured in liquid PDA medium for 48 h and then washed three times to remove the medium. The relative conductivity (RC) of liquid-culture hyphae under the treatment of W10-Sp1 protein (two different concentrations were used) or sterile water was measured at 2, 4, 6, 12, and 24 hpi. Treatment of W10-Sp1 protein with nine kinds of fungi significantly increased the conductivity in the solutions in addition to *G. graminis*; the higher the Sp1 protein concentration was used, the higher the value of RC (Figures 5A–H). The results indicated that cell structure of the fungi was damaged under W10-Sp1 protein treatment.

### W10-Sp1 protein was involved in activating systemic resistance

**FIGURE 6:**
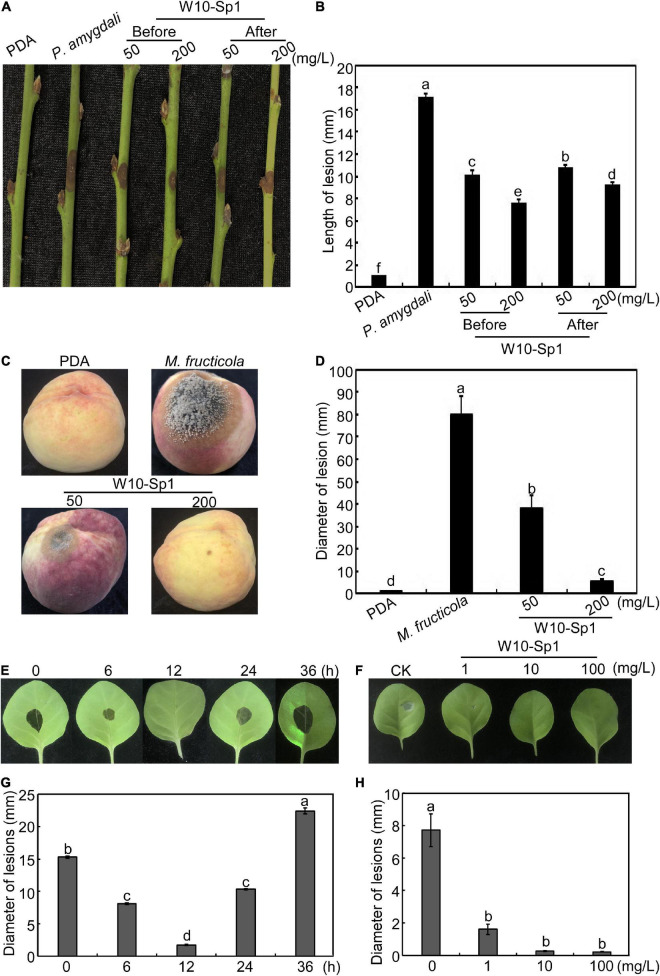
W10-Sp1 activated systemic resistance of the host. **(A)** Two treatments for control of peach shoot blight: hyphal cakes of *P. amygdali* were inoculated on the wounded peach twigs for 24 h, after which the hyphal cakes were removed and the twigs were dipped in the W10-Sp1 protein solution for 30 min (after); another treatment used W10-Sp1 protein solution to dip the disinfected twigs for 30 min, and then the hyphal cakes of *P. amygdali* were inoculated (before). The results of both treatments were observed at 7 dpi. **(B)** Measurement of lesion length at 7 dpi for peach shoot blight-control effect. **(C)** W10-Sp1 protein solution was sprayed onto disinfected peach fruits for 30 min at different concentrations, and then the hyphal cakes of *M. fructicola* were inoculated, and the results were observed at 7 dpi. **(D)** Measurement of lesion diameters at 7 dpi for peach brown rot-control effect. Direct inoculation of hyphal cakes was used as a positive control; equal solid PDA media inoculation was used as a negative control. **(E)** W10-Sp1 protein was injected into the leaves of Xanthi tobacco and inoculated with hyphal cakes of *B. cinerea* on upper leaves at 0, 6, 12, 24, and 36 h post-injection (hpi), and disease symptoms were observed at another 24 hpi. **(F)** Measurement of lesion diameters at 24 hpi of different incubation time treatments of Sp1 protein. **(G)** Different concentrations of purified Sp1 protein were injected into Xanthi tobacco and then inoculated into hyphal cakes of *B. cinerea* at 12 h post-injection. CK was treated with equal amounts of sterile water. **(H)** Measurement of lesion diameters at 24 hpi after treatment with different concentrations of Sp1 protein.

Based on the significant antifungal effect of the W10-Sp1 protein, we wanted to explore its effect on disease control. Hyphal cakes of *P. amygdali* were inoculated onto disinfected and wounded peach twigs and then dipped into 50 mg/L or 200 mg/L W10-Sp1 protein for 30 min at 24 hpi. Inoculated PDA cakes or hyphal cakes without Sp1 treatment were used as negative or positive controls, respectively, and disease symptoms were observed at 7 dpi. The length of lesions were reduced by 36.8∼46.1% compared with no treatment with W10-Sp1 protein (Figures 6A,B). Furthermore, disinfected peach twigs were first dipped into the same concentration of W10-Sp1 protein for 30 min and then inoculated with hyphal cakes of *P. amygdali*. Symptoms were observed at 7 dpi. Amazingly, the length of lesions were reduced by 40.9∼55.5%, and the peach shoot blight-control effect was better than that of treatment with W10-Sp1 solution after inoculation (Figures 6A,B). In addition, hyphal cakes of *M. fructicola* were inoculated on peach fruits after spraying different concentrations of W10-Sp1 protein solution and cultured at 28°C with 90% relative humidity for 7 days. PDA-treated peach fruits and only-inoculated hyphal cakes were used as controls. The results showed that the peach brown rot-control rate was reduced by 52.4% under the treatment of 50 mg/L W10-Sp1 protein and 93.5% under the treatment of 200 mg/L W10-Sp1 protein (Figures 6C,D).

Furthermore, 10 mg/L W10-Sp1 protein was injected into the lower leaves of Xanthi tobacco, and hyphal cakes of *B. cinerea* were inoculated onto the upper leaves at different induction times from 0∼36 hpi. The results showed that the best induced gray mold resistance time by the W10-Sp1 protein was at 12 hpi, and the lesion diameters were approximately 2.5 mm (Figures 6E,F). Furthermore, different concentrations of 1, 10 or 100 mg/L W10-Sp1 protein were applied to the lower leaves of Xanthi tobacco for 12 h, after which *B. cinerea* was inoculated onto the upper leaves, and symptoms were observed at 24 hpi, separately. The results showed that the diameters of the lesions were 1.62, 0.28 and 0.23 mm at different inducing concentrations. There was no significant differences among the three concentrations (Figures 6G,H). Overall, the results revealed that the W10-Sp1 protein activated systemic resistance independent of its concentration.

### Transcriptome analysis of W10-Sp1 protein activation systemic resistance mechanism

**FIGURE 7:**
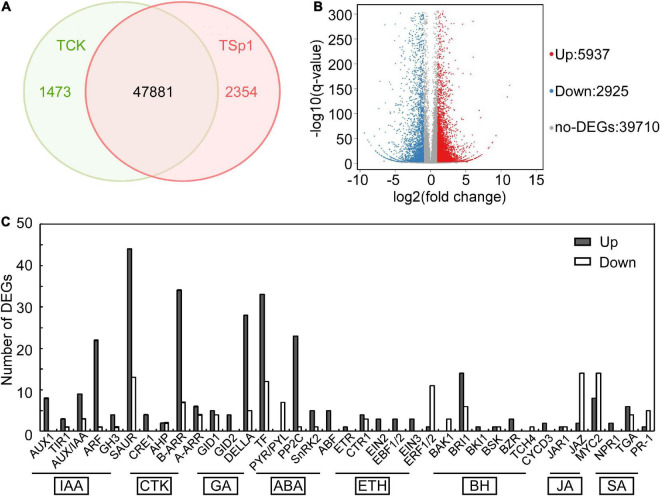
Transcriptome analysis between the samples of W10-Sp1 treating Xanthi tobacco (TSp1) to Xanthi tobacco (TCK). **(A)** Venn diagram presenting the overlap of expressed genes between TSp1 and TCK. Green represents the number of expressed genes of TCK; red represents the number of expressed genes of TSp1. **(B)** Volcano plot of DEGs between TCK and TSp1. Splashes represent different genes, where red indicates significantly upregulated genes after Sp1 protein was used to treat Xanthi tobacco, blue indicates downregulated genes, and gray indicates no significant difference. **(C)** Analysis of DEGs focusing on the plant hormone signaling pathway of TSp1 vs. TCK. The gray box represents upregulated DEGs of TSp1 vs. TCK, and the white box represents downregulated DEGs.

W10-Sp1 protein-treated Xanthi tobacco (TCK) was used to further explore its mechanism of systemic resistance through transcriptome analysis due to the complete genome information of tobacco. The RNA of sterile water-treated Xanthi tobacco (TSp1) was used as a control group. RNA-seq was verified by qRT-PCR by selecting 16 genes involved in auxin synthesis, SA metabolism, JA metabolism and phenylpropanoid biosynthesis. The expression trends of qRT-PCR were consistent with those of RNA-seq in addition to that of *CCOAOMT2*. Thus, the RNA-seq had approximately 93.8% accuracy (Supplementary Figure S4). A total of 51,708 transcripts were obtained, among which 47,881 were present in both conditions. However, 1473 and 2354 transcripts were specifically expressed in TCK or TSp1 samples, respectively (Figure 7A). DEG analysis found that 5937 transcripts were upregulated (FC ≥ −2, *q*-value ≤ 0.001), 2925 transcripts were downregulated (FC ≤ −2, *q*-value ≤ 0.001), and most of the genes (39,710 transcripts) had no differential expression (FC < 2 or *q*-value > 0.001) (Figure 7B). The average total numbers of clean reads were 44.91 Mb and 44.74 Mb; the Q20 values were 99.02% and 99.04%; the Q30 values were 96.89% and 96.95%; and the total mapping ratios were 85.06% and 85.18% in the three TCK and TSp1 samples, respectively (Supplementary Table S2). The top 10 GO enrichments were metabolic process, cellular process, binding, catalytic activity, cell, cell part, membrane, organelle, membrane part and response to stimulus (Table 3). The top 10 KEGG pathways enriched were metabolic pathway, biosynthesis of secondary metabolites, plant hormone signal transduction, plant–pathogen interaction, MAPK signaling pathway-plant, protein processing in endoplasmic reticulum, starch and sucrose metabolism, biosynthesis of amino acids, phenylpropanoid biosynthesis, and endocytosis (Table 4).

**TABLE 3:** The top 10 GO enrichment list of 5618 DEGs of Sp1 protein vs. control tobacco.

Ontology	GO term	Number of genes
biological_process	Metabolic process	805
biological_process	Cellular process	721
molecular_function	Binding	700
molecular_function	Catalytic activity	689
cellular_component	Cell	597
cellular_component	Cell part	590
cellular_component	Membrane	454
cellular_component	Organelle	411
cellular_component	Membrane part	388
biological_process	Response to stimulus	263

**TABLE 4:** The top 10 KEGG pathway functional enrichment results of Sp1 protein –vs.- control tobacco.

Pathway	DEGs genes with pathway annotation (6880)	All genes with pathway annotation (51507)	*P*-value	*Q*-value	Pathway ID
Metabolic pathways	1709 (24.84%)	13255 (25.73%)	0.967253	1.0	ko01110
Biosynthesis of secondary metabolites	995 (14.46%)	7021 (13.63%)	0.01668	0.09789	ko01110
Plant hormone signal transduction	407 (5.92%)	1965 (3.82%)	0	0	ko04075
Plant–pathogen interaction	364 (5.29%)	2921 (5.67%)	0.93336	1.0	ko04626
MAPK signaling pathway–plant	308 (4.48%)	2117 (4.11%)	0.05460	0.26321	ko04016
Protein processing in endoplasmic reticulum	261 (3.79%)	2313 (4.49%)	0.99901	1.0	ko04141
Starch and sucrose metabolism	235 (3.42%)	1487 (2.89%)	0.00326	0.02591	ko00500
RNA transport	213 (3.1%)	1853 (3.6%)	0.99337	1.0	Ko03013
Phenylpropanoid biosynthesis	205 (2.98%)	1762 (3.42%)	0.98720	1.0	ko00940
Endocytosis	181 (2.63%)	1845 (3.58%)	1.0	1.0	ko04144

### The W10-Sp1 protein significantly affected plant hormones, especially increasing the intracellular contents of salicylic acid and jasmonic acid

Plant hormones play important roles in various stress conditions, including biotic or abiotic stress (Coolen et al., 2016). RNA-seq in the samples of W10-Sp1 protein-treated Xanthi tobacco also significantly induced plant hormone signal transduction, and approximately two-thirds were upregulated. Nearly all genes that regulated auxin (IAA), cytokinin (CTK), gibberellin (GA), abscisic acid (ABA), and salicylic acid (SA) signal transduction were differentially expressed. In total, 96 DEGs were upregulated and 34 DEGs were downregulated in IAA signal transduction; 46 DEGs were upregulated and 13 DEGs were downregulated in CTK signal transduction; 70 DEGs were upregulated and 21 DEGs were downregulated in GA signal transduction; and 33 DEGs were upregulated and 9 DEGs were downregulated in ABA signal transduction (Figure 7C).

**FIGURE 8:**
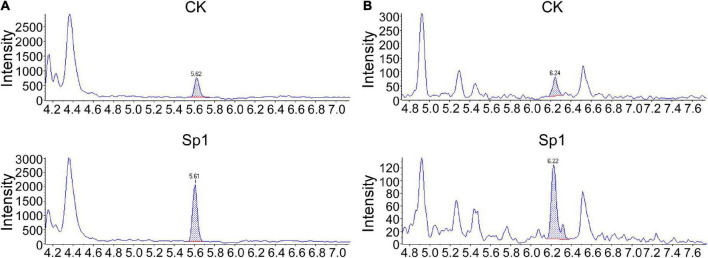
MS/MS spectra of MRM chromatograms of detected the contents of SA and JA in 50 mg of the Xanthi tobacco samples treated with W10-Sp1 protein or control. **(A)** MS/MS spectra of detected SA from 50 mg of upper leaves of Xanthi tobacco under different treatments. The peak flow of the standard of SA was at ∼5.61 min. **(B)** MS/MS spectra of detected JA from 50 mg of upper leaves of Xanthi tobacco under different treatments. The peak flow of the standard of JA was at ∼6.22 min.

In addition, plant hormones such as SA and JA are important regulators of activating systemic resistance; therefore, DEGs in the SA and JA signal transduction pathways were further analyzed by RNA-seq. NPR1, a positive regulator of the SA signaling pathway, was upregulated by two genes. There were six upregulated and four downregulated *TGA* genes. However, the disease resistance-related gene *PR1* was upregulated by one gene and downregulated by five genes, which showed more significant differences. In the JA signaling pathway, the negative regulatory factor *JAZ* was upregulated by 2 genes and downregulated by 14 genes with more significant differences. There were 8 upregulated and 14 downregulated genes in the transcription factor *MYC2* that regulated JA signal termination, and downregulated genes showed more significant differences, suggesting that W10-Sp1 might enhance the JA signaling pathway in Xanthi tobacco by inhibiting the expression of its negative regulatory genes (Supplementary Table S3). These results indicated that the SA and JA signaling pathways were induced by the W10-Sp1 protein. Furthermore, the contents of SA and JA were detected in different treated samples with HPLC-MS/MS method. The content of SA increased 7.2-fold and that of JA increased 4.8-fold compared W10-Sp1-treated Xanthi tobacco for 12 h to untreated tobacco in the upper leaves of Xanthi tobacco (Figure 8 and Table 5). The results suggested that W10-Sp1 treatment activated the intracellular SA and JA signaling pathways.

**TABLE 5:** The contents of SA and JA in the samples of W10-Sp1-treated Xanthi tobacco for 12 h and untreated tobacco with HPLC-MS/MS method.

Hormone	TCK (ng/g)	TSp1 (ng/g)
SA	0.3106 ± 0.0148	2.2312 ± 0.0990**
JA	0.0183 ± 0.0049	0.0879 ± 0.0192**

The content of each hormone component in the sample (ng/g) = The concentration of each component in the test solution (ng/ml) × Sample extraction liquid volume (ml)/The sample quality (g). Asterisks represent a significant differences. Experiments were repeated three times with similar results. Duncan’s new multiple range test, p < 0.01.

### Impact of W10-Sp1 protein on the plant–pathogen interaction pathway of tobacco

**FIGURE 9:**
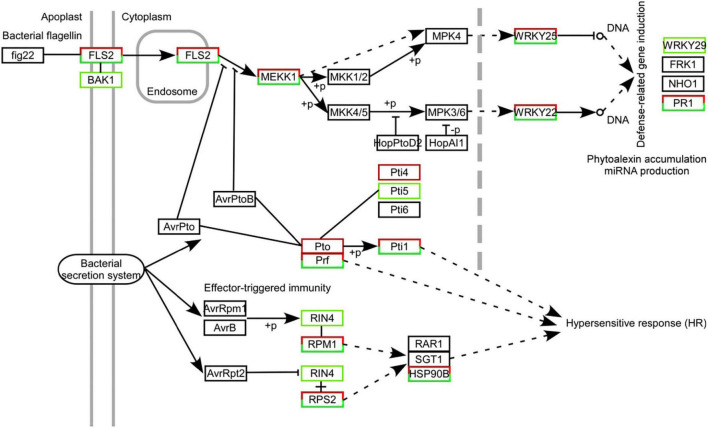
Statistical map of DEGs in the immune signaling pathways mediated by Fls2, Prf, and Rpm1 strongly induced in Xanthi tobacco treated with the W10-Sp1 protein. The black box represents no differential expression; the red box represents DEGs with upregulation; the green box represents DEGs with downregulation; and the red and green combined box represents both upregulated and downregulated DEGs.

A total of 426 DEGs were identified in the plant–pathogen interaction pathway under W10-Sp1 treatment, including Ca^2+^/CaM signal pathway-related genes (*NOS*, *CML*), the transcription factor WRKY, serine/threonine-protein kinases (*PTO*, *PBS1*), and a disease resistance protein (*RPS4*) (Figure 9 and Supplementary Table S4). Furthermore, the plant–pathogen interaction pathway that was potentially regulated by W10-Sp1 was described according to the number of DEGs. We found that family genes of *RPM1*, which recognized bacterial effectors, included 81 upregulated DEGs and 8 downregulated DEGs; LRR receptor-like serine/threonine-protein kinase *FLS2*, which recognized bacterial flagellin signaling transduction pathways, was upregulated by 66 genes and downregulated by 18 genes. In addition, recognition of bacterial effector signal transduction pathway disease resistance protein Prf family genes showed 44 upregulated genes and 2 downregulated genes, and other genes in these pathways were also differentially upregulated or downregulated (Figure 9). Further, the expressions of receptor-genes were significantly up-regulated or down-regulated by qRT-PCR (Supplementary Figure S5). These results indicated that W10-Sp1 treatment might induce the plant defense response to control pathogen infection by significantly stimulating the expression of these three signaling pathways.

## Discussion

Our previous work purified and identified a serine protease, Sp1, from *B. licheniformis* W10 strains, which significantly inhibited the growth of *B. cinerea* and had higher temperature and pH tolerance, suggesting that the protein could be a potential biopesticide (Ji et al., 2020b). To explore its applicability, here, the purified W10-Sp1 protein notably inhibited the growth of ten kinds of plant pathogenic fungi (*B. cinerea, P. amygdali*, *R. solani*, *M. fructicola*, *S. sclerotiorum*, *G. cingulata*, *R. stolonifer*, *F. moniliforme*, *A. cerasi* and *G. graminis*) and four kinds of bacteria (*X. arboricola*, *P. cichorii*, *P. syringae*, *A. avenae*), indicating that the W10-Sp1 protein has a broad antimicrobial spectrum. Most of the literature have reported that proteins secreted by *Bacillus* have antifungal activity; however, little is known about their antibacterial activity (Li et al., 2009; Cui et al., 2012; Wang et al., 2014; Slimene et al., 2015; Yan et al., 2021). It was not clear whether the antimicrobial spectra of different antifungal proteins were consistent, and some pathogenic fungi were not detected.

In this work, the antifungal activity of the W10-Sp1 protein might be the reason for its affecting hyphal structure (including increasing hyphal diameter, leading to swollen hyphae and producing vesicular-like structures) and inhibiting spore germination. The characteristics were in accordance with those of the treatment of *B. licheniformis* W10 strains (Tang et al., 2005; Sun et al., 2007), which indicated that the W10-Sp1 protein was an important extracellular secreted substance for the biocontrol function of *B. licheniformis*. Furthermore, the EC_50_ values of its antifungal activity were 4.2–398.2 mg/L with 10 different plant pathogenic fungi; among them, the W10-Sp1 protein had a much better biocontrol effect on *G. graminis*, and that of *B. cinerea* was the worst. The EC_50_ values provide an important experimental basis for the applicability of the products of the W10-Sp1 protein as the main ingredients in the future.

**FIGURE 10:**
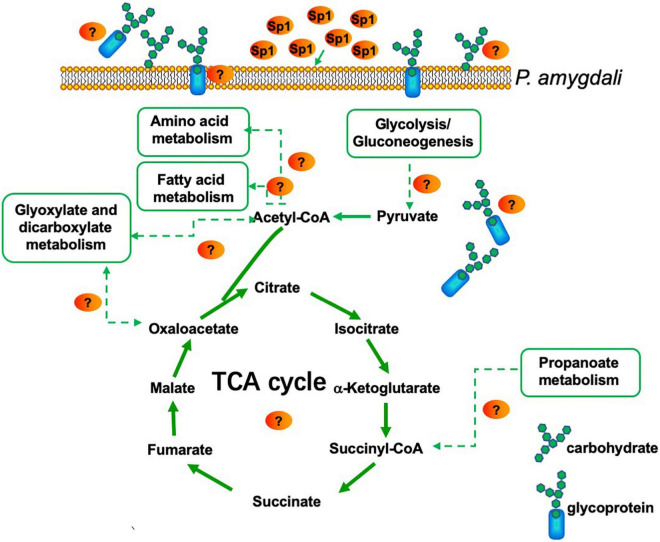
Model for *P. amygdali* inducing energy supply and cell wall structure by purified W10-Sp1 protein.

In addition, *P. amygdali*, which causes peach shoot blight to seriously affect peach production (Yang et al., 2022), was used as an example in the present work to explore the underlying molecular regulatory mechanism of the W10-Sp1 protein in plant pathogenic fungi through transcriptome analysis. RNA-seq identified 359 DEGs, 150 of which were upregulated and 209 of which were downregulated in the whole genome of *P. amygdali* treated with the W10-Sp1 protein when compared with untreated hyphae of *P. amygdali* (PCK). The top 10 GO enrichment and KEGG pathway enrichment analyses showed that treatment of *P. amygdali* with the W10-Sp1 protein significantly affected coenzyme binding, carbohydrate metabolic processes, amino acid metabolism, fatty acid metabolism, glyoxylate and dicarboxylate metabolism, and propanote metabolism. Interestingly, those pathways and biological processes had a common intersection, which was the TCA cycle. DEGs in the molecular function category of coenzyme binding, such as acetyl-CoA, which is a key regulator of the TCA cycle, were highly active. Amino acids are indispensable components in the formation of cell structures and various enzymes. The degradation of valine, leucine and isoleucine might be related to propanoate metabolism, which enters the TCA cycle (Tian et al., 2021). DEGs of valine, leucine and isoleucine degradation were all upregulated in this work, indicating that some cell structures and enzymatic activities might be damaged, similar to the hyphal morphological changes in *P. amygdali*. The results suggested that W10-Sp1 treatment affected the energy supply and amino acid metabolism of *P. amygdali*. In addition, genes expressing glycosyl hydrolase (GME11525_g, GME3789_g, GME4018_g, GME13604_g, GME7161_g, GME3329_g, GME10136_g) and glucanase (GME6808_g, GME4201_g, GME10462_g) were notably downregulated. Protein glycosylation is one of the most common and highly conserved posttranslational modifications in eukaryotes and plays important roles in protein structure and function (Willer et al., 2005). It has been reported that glycosylation is closely related to the virulence of plant pathogens and affects biological processes such as pathogen adhesion, signal transduction, immune response and colonization (Li et al., 2013; Rempe et al., 2016; Liu et al., 2021). Among them, glycosyl hydrolase and glycosyltransferase drive essential functions. Glycosylated proteins play important roles in composition of fungal cell wall structure. Moreover, glycolysis and the TCA cycle are both important energy supply processes, and there is crosstalk between the two processes (Payne et al., 2010). Besides that, previous work has reported that cyclic lipopeptides isolated from *Bacillus* inhibited the activity of (1,3)-beta-D-glucan synthase in fungi and caused abnormal cell walls (Kurtz and Douglas, 1997). They all affected the composition of the cell wall. Therefore, we suspect that the W10-Sp1 protein might affect the energy supply and cell wall structure, to inhibit the development of *P. amygdali* (Figure 10). Conductivity analysis confirmed the results that W10-Sp1 treatment surely damaged the cell wall of nine fungi. To our knowledge, this is the first study to uncover how biocontrol antifungal proteins protect against plant pathogens.

Furthermore, we found that the W10-Sp1 protein has a better disease control effect, especially when pre-spraying the host, than when the protein was sprayed after inoculation with the pathogen. Furthermore, injection into the lower leaf of Xanthi tobacco within Sp1 protein for 12 h significantly inhibited the infection of *B. cinerea*, indicating that W10-Sp1 activated systemic resistance of the host, including peach twigs, fruits and Xanthi tobacco. Transcriptome analysis in Xanthi tobacco treated or not treated with the W10-Sp1 protein was used to explore the underlying mechanism of systemic resistance. There were 8862 DEGs treated with W10-Sp1 protein for 12 h compared with sterile water-treated Xanthi tobacco, 5937 of which were upregulated and 2925 of which were downregulated. The DEGs were mainly enriched in five pathways, including metabolic pathways, biosynthesis of secondary metabolites, plant hormone signal transduction, plant–pathogen interaction and MAPK signaling pathway–plant. The pathogenesis of plant–pathogen interactions and plant hormone signal transduction were also induced by hosts infected with pathogens such as sunflower/tomato infected with *Verticillium dahlia* (Tan et al., 2015; Guo et al., 2017) or biocontrol bacteria pre-treated with hosts and then infected with pathogens, such as cotton inoculated with *Chaetomium globosum* CEF-082 and *V. dahlia* (Zhang et al., 2020).

Plants encounter a variety of microorganisms, including both beneficial and pathogenic microorganisms, which establish an interaction relationship with each other through their sophisticated innate immune response (Rodriguez et al., 2019). In general, beneficial microorganisms can activate induced systemic resistance (ISR) of plants to suppress plant disease in uninoculated plant organs, and pathogenic microorganisms activate systemic acquired resistance (SAR) of plants, thus relieving plant disease in uninoculated plant organs (Nishad et al., 2020). Plant hormones, including SA and JA/ET, are well known to activate plant defense response (Robert-Seilaniantz et al., 2011; Pieterse et al., 2012); among them, SA signaling is vital for SAR (Bernsdorff et al., 2016); however, JA/ET signaling plays important roles in ISR independent of the SA signaling pathway (Ahn et al., 2007). Most work has demonstrated that antagonistic microorganisms or their secondary metabolites can activate ISR (Shoresh et al., 2010; Elsharkawy et al., 2013; Mejri et al., 2018). In the present study, transcriptome analysis found that DEGs in the SA and JA signaling pathways were highly active, and the contents of intracellular SA and JA were measured, which both significantly increased with W10-Sp1 treatment of Xanthi tobacco, implying that the Sp1 protein activated SAR and ISR in tobacco by simultaneously activating the SA and JA signaling pathways. The results were in agreement with the study that showed that *B. cereus* AR156 activated SA and JA signaling to activate systemic resistance in *Arabidopsis thaliana*, and similar results were obtained in *P. fluorescens* WCS417 in tomato against *P. syringae* pv. *tomato* and *B. amyloliquefaciens* I3 in the seeds of *A. thaliana* (Van Wees et al., 1997; Niu et al., 2011; Ilham et al., 2019). This might be the first report indicating that systemic resistance induced by antifungal proteins shares features of SAR and ISR.

In addition, RNA-seq in this study identified that DEGs were also enriched in the plant–pathogen interaction pathway and found that certain genes, including *CDPK, RBOH, CNGCS, FLS2, PTO, PRF, NOS, PR-1, PBS1* and *CML*, were significantly upregulated or downregulated. A further statistical map of DEGs indicated that the W10-Sp1 protein strongly activated the Fls2-, Prf- and Rpm1-mediated immune response pathways, which might trigger the defense response of Xanthi tobacco, thus producing disease resistance. The immune responses mediated by Fls2, Rboh, CNGC and CaM/CML were activated after plants were infected by pathogens or plants were treated with biocontrol bacteria or exposed to various stresses in previous work (Zhang et al., 2014, 2020; Singh and Bhatla, 2016).

There is a close relationship between plant development and disease resistance. RNA-seq of TSp1 to TCK identified that DEGs were enriched in photosystem, protein chromophore, chlorophyll and defense response in the GO lineage DAG graph (Supplementary Figure S6), indicating that W10-Sp1 protein might promote the growth of Xanthi tobacco by strengthening its photosynthesis. In addition, the plant hormones IAA, GA, and CTK were differentially expressed, which also affected the development of Xanthi tobacco treated with the W10-Sp1 protein. It is worth mentioning that *B. licheniformis* W10 is a plant growth-promoting rhizobacterium (PGPR). These results indicated that the Sp1 protein was surely an important antimicrobial protein of the W10 strain.

In summary, in the present work, we found that serine protease Sp1 secreted by *B. licheniformis* W10 has a broad antifungal spectrum by inhibiting its vegetative growth, changing its hyphal structure and reducing the spore germination. In addition, the W10-Sp1 protein also has an inhibitory effect on some plant pathogenic bacteria. Furthermore, dual transcriptome analysis indicated its potential molecular mechanism of anti-fungal resistance, activated systemic resistance and induced plant defense responses. The W10-Sp1 protein might perform similar functions in a variety of plant pathogenic fungi and plants. This study supplies a solid foundation for the applicability of the Sp1 protein.

## Data availability statement

The raw data of RNA-seq in this work has successfully submitted to Sequence Read Archive (SRA) database at National Center for Biotechnology Information (NCBI). The BioProject ID was PRJNA852429 (https://www.ncbi.nlm.nih.gov/search/all/?term=%20PRJNA852429) and PRJNA853397 (https://www.ncbi.nlm.nih.gov/search/all/?term=+PRJNA853397).

## Author contributions

LY, LL, and ZJ designed, supervised, and wrote the manuscript. CY, JG, and YL performed the experiments and analyzed the data. SP and LC supplied the transcriptome analysis. All authors have read and approved the manuscript.

## Supplementary material

Supplementary Figure S1 Identification of purified W10-Sp1 protein by SDS-PAGE.

Supplementary Figure S2 Effects of different concentrations of W10-Sp1 protein to the mycelium of 10 kinds of plant pathogenic fungi.

Supplementary Figure S3 Verification of RNA-Seq of PSp1-vs.- PCK by qRT-PCR.

Supplementary Figure S4 Verification of RNA-Seq of TSp1-vs.- TCK by qRT-PCR.

Supplementary Figure S5 Directed acyclic graph (DAG) of GO enrichment of DEGs in Xanthi tobacco treated by W10-Sp1 protein.
