# Supplementary material for: Broad-spectrum resistance mechanism of serine protease Sp1 in Bacillus licheniformis W10 via dual comparative transcriptome analysis
Source: Front Microbiol. 2022 Oct 4;13:974473. doi: 10.3389/fmicb.2022.974473 (PMC9577198; doi:10.3389/fmicb.2022.974473)
Supplement: Supplementary file 1 [file Table_1.docx]

Table S1 Clean read quality metrics of *P. amygdali* ZN32 strain (PCK) and purified W10-Sp1-treated *P. amygdali* ZN32 strain (PSp1).

| Sample | Total Clean Reads (Mb) | Clean Reads Q20 (%) | Clean Reads Q30 (%) | Clean Reads Ratio (%) | Total Mapping Ratio (%) |
| --- | --- | --- | --- | --- | --- |
| PCK-1 | 43.54 | 99.03 | 97.88 | 97.73 | 97.98 |
| PCK-2 | 48.09 | 99.01 | 97.87 | 93.78 | 98.00 |
| PCK-3 | 44.51 | 99.03 | 97.83 | 93.65 | 97.85 |
| PSp1-1 | 41.77 | 99.08 | 97.84 | 93.65 | 97.97 |
| PSp1-2 | 44.45 | 99.05 | 97.82 | 93.62 | 98.10 |
| PSp1-3 | 41.23 | 99.00 | 98.01 | 94.01 | 98.13 |

Total Clean Reads represent the reads amount after filtering; Clean Reads Q20 represent the Q20 value for the clean reads; Clean Reads Q30 represent the Q30 value for the clean reads; Clean Reads Ratio represent the ratio of the amounts of clean reads; Total Mapping Ratio represents the percentage of mapped reads.
