# Supplementary material for: Broad-spectrum resistance mechanism of serine protease Sp1 in Bacillus licheniformis W10 via dual comparative transcriptome analysis
Source: Front Microbiol. 2022 Oct 4;13:974473. doi: 10.3389/fmicb.2022.974473 (PMC9577198; doi:10.3389/fmicb.2022.974473)
Supplement: Supplementary file 2 [file Table_2.docx]

Table S2 Clean read quality metrics of Xanthi tobacco (TCK) and purified W10-Sp1 treating Xanthi tobacco (TSp1).

| Sample | Total Clean Reads (Mb) | Clean Reads Q20 (%) | Clean Reads Q30 (%) | Clean Reads Ratio (%) | Total Mapping Ratio (%) |
| --- | --- | --- | --- | --- | --- |
| TCK-1 | 45.04 | 99.03 | 96.89 | 92.72 | 85.53 |
| TCK-2 | 44.71 | 99.01 | 96.86 | 92.03 | 84.17 |
| TCK-3 | 44.98 | 99.03 | 96.93 | 92.60 | 85.48 |
| TSp1-1 | 45.18 | 99.08 | 97.05 | 93.00 | 87.27 |
| TSp1-2 | 44.85 | 99.05 | 96.95 | 92.34 | 85.44 |
| TSp1-3 | 44.20 | 99.00 | 96.85 | 90.98 | 82.83 |

Total Clean Reads represent the reads amount after filtering; Clean Reads Q20 represent the Q20 value for the clean reads; Clean Reads Q30 represent the Q30 value for the clean reads; Clean Reads Ratio represent the ratio of the amounts of clean reads; Total Mapping Ratio represents the percentage of mapped reads.
