# Supplementary material for: Broad-spectrum resistance mechanism of serine protease Sp1 in Bacillus licheniformis W10 via dual comparative transcriptome analysis
Source: Front Microbiol. 2022 Oct 4;13:974473. doi: 10.3389/fmicb.2022.974473 (PMC9577198; doi:10.3389/fmicb.2022.974473)
Supplement: Supplementary file 3 [file Table_3.docx]

Table S3 Statistical analysis of DEGs in JA and SA signalling pathway after W10-Sp1 protein treating Xanthi tobacco.

| Gene name | Change type | DEGs Number | Gene ID and log_2_(Fold change) |
| --- | --- | --- | --- |
| **JA signal pathway** | | | |
| *JAR1* | Up | 1 | 107761031 (1.7) |
|  | Down | 1 | 107772988 (-1.8) |
| *JAZ* | Up | 2 | 107785869 (1.2)、107761032 (1.1) |
|  | Down | 14 | 107794802 (-5.1)、107769557 (-5.0)、107831139 (-4.9)、 107798011 (-4.9)、107801671 (-4.2)、107815661 (-3.5)、 107763851 (-2.7)、107808301 (-2.5)、107798038 (-2.0)、 107798531 (-1.5)、107763941 (-1.2)、107767880 (-1.2)、 107766083 (-1.0)、BGI_novel_G, 000282 (-2.1) |
| *MYC2* | Up | 8 | 107807794 (2.7)、107820556 (1.8)、107810230 (1.7)、 107775911 (1.7)、107824258 (1.4)、107764381 (1.4)、107815499 (1.4)、107826976 (1.0) |
|  | Down | 14 | 107825229 (-6.7)、107811233 (-6.1)、107811232 (-5.7)、 107825228 (-4.0)、107801711 (-3.3)、107805377 (-3.1)、 107766344 (-2.5)、107765893 (-2.5)、107774268 (-1.9)、 107769417 (-1.9)、107824270 (-1.9)、107782755 (-1.9)、 107774314 (-1.7)、107774553 (-1.2) |
| **SA signal pathway** | | | |
| *NPR1* | Up | 2 | 107820609 (2.0)、107811716 (1.1) |
| *TGA* | Up | 6 | 107813754 (4.4)、107808735 (3.0)、107790481 (1.9), 107772044 (1.3)、107815287 (1.3)、BGI_novel_G003104 (2.3) |
|  | Down | 4 | 107771305 (-2.7)、107782927 (-2.6)、107768553 (-1.7)、 107775514 (-1.2) |
| *PR-1* | Up | 1 | 107791665 (3.9) |
|  | Down | 5 | 107808770 (-9.4)、107807832 (-8.9)、107763263 (-7.8)、 107768378 (-5.1)、107798618 (-4.8) |
