# Supplementary material for: Broad-spectrum resistance mechanism of serine protease Sp1 in Bacillus licheniformis W10 via dual comparative transcriptome analysis
Source: Front Microbiol. 2022 Oct 4;13:974473. doi: 10.3389/fmicb.2022.974473 (PMC9577198; doi:10.3389/fmicb.2022.974473)
Supplement: Supplementary file 4 [file Table_4.docx]

Table S4 Statistical analysis of DEGs in plant-pathogen interaction pathway after W10-Sp1 protein treating Xanthi tobacco.

| Change type | Number | Gene name of KEGG annotation |
| --- | --- | --- |
| Up-regulated expression | 269 | *CDPK、Rboh、CNGCS、NOS、FLS2、MEKK1、WRKY25、WRKY22、PR-1、EFR、Pto、Prf、Pti4、Pti1、RPM1、RPS2、PBS1、HSP90B、RPS4、EDS1、WRKY1* |
| Down-regulated expression | 157 | *CDPK、Rboh、CNGCS、CML、FLS2、BAK1、MEKK1、WRKY25、WRKY29、WRKY22、PR-1、EFR、Prf、Pti5、Pti1、RIN4、RPM1、RPS2、PIK1、HSP90、EDS1、RRS1-R、HCD1、WRKY1* |
