# Supplementary material for: Broad-spectrum resistance mechanism of serine protease Sp1 in Bacillus licheniformis W10 via dual comparative transcriptome analysis
Source: Front Microbiol. 2022 Oct 4;13:974473. doi: 10.3389/fmicb.2022.974473 (PMC9577198; doi:10.3389/fmicb.2022.974473)
Supplement: Supplementary file 5 [file Table_5.docx]

Table S5 qRT primers used in this study.

| Gene/Gene ID | Accession | F primer (5’→3’) | R primer (5’→3’) |
| --- | --- | --- | --- |
| *PaH3* | KC343503 | CGTCGCCCCTCCCCACAGGCATG | CCGATGGCGGAGGACTGGAAG |
| *GME1740_g* | P53395 | GTCTTCTGGTGCGTCTGA | AGTGTCCATTGCTATCCC |
| *GME8443_g* | Q9DBL1 | ACCTCCTATCACTACTGCC | GACTAAACCACCAACCAA |
| *GME6808_g* | D4B0V1 | GCGGATTGTTTATCTTTGTT | GAGTCACAGCCCTTTCCA |
| *GME10462_g* | Q2UPQ4 | TGGTCCTGAACCTCAACC | CCATCCGAGTCCAAATAC |
| *GME2926_g* | P15368 | GCACGGTGACTTCTACGC | CACTGGCTGACAATTTCG |
| *GME8738_g* | Q6AZA0 | TATCCCAGCGACTCCTAT | TCCACGCCTTACACTATG |
| *GME3036_g* | P0CJ44 | AAAGTGAGACGGAAGGAAC | AAGGCTATTGCCGAGATT |
| *GME2854_g* | C8VP36 | ACCTTGTTATGCCGCTACT | CAAATCCGCAATCTCCTC |
| *EF-1α* | AF120093 | CTCAGTCACCCAAGCCCTCA | CGTTCCAGCCCTTACCACAT |
| *LAX2* | XP_009798935 | TGGGTTTGGATTTGGTGGTTGGG | GTGGTGGGGTGGGGAATTGTTTAG |
| *AUX22* | XP_009623794 | CGAAGCGGTGAAAGATGGAGACAG | GAACGTCGCCGAGAAGCATCC |
| *GH3.6* | XP_009631635 | CCTGACCCAGAGTTGGCTGATTTC | ATACTGCGACATGCTTCCGGTTAC |
| *AX10A* | XP_009799980 | CGGTCTCTTTCTGTTGCCGG | CCACACCCTAAAGCTTTGGCA |
| *TGA2* | XP_009613713 | AACGCTGCTTCCTTTGGATGGG | GTGCTTCTTCTGCTTGTTGCGATG |
| *PR-1* | XP_009606837 | TGCAAAATGGTGGGCAAGTTCAAG | CAGCCCAAGCATTAACAGCATCTG |
| *HBP-1b* | XP_009623095 | AAGAAACAGAAGAGGGTG | AACCGAATCAGTAACCAG |
| *NPR5* | XP_009799339 | ATCGCTAATGCCACACCATCACG | AGTCAAGGGCTCGTCTCATCCG |
| *TIFY 5A* | XP_009622676 | AGCTCCATTCATTCGCAGCT | GCCTGAGCTCCAAGTTACAGTT |
| *TIFY10A* | XP_009759848 | TTGCGTAGCTGGTGAAGTTACTGC | GCTTCTGCTATGTGCGGAGTCC |
| *Bhlh18* | XP_009605673 | GTCATCGGCTACATGGTGGTCTG | CGGTCATCGTCTGGCAGTGTTC |
| *Bhlh041* | XP_009621466 | AGGAGGCAAGAACAGATTCAGGTG | TCCAGGAGGGAGCAAAGATCTCAG |
| *POD16* | XP_009600643 | GCCTGTTGCTAAGATTGG | GTTACTGTGCTCGTTGCT |
| *8HGO* | XP_009764992 | CGCTCAGCCCATTCTTAT | TTTCCTCCGTCACATCAC |
| *GT2* | XP_009790173 | CATTCCCAGGCCAAGGTC | ATCCCAAGCATCATCAAAGA |
| *CCOAOMT2* | XP_009604110 | GGACGCTGACAAAGACAA | CGAAATCCCTATAATACCTAAC |
| *FLS2* | XP_107781359 | GTTTCACAGGCCCAATTCCTG | TACCAATTTCTTCAGGAATGA |
| *BAK1* | XP_107825933 | GGATGAGGAGTTTGAAGCAGT | TCCCTTCACCCAATCAAGCAAC |
| *PRF* | XP_107783738 | GACTAAGTGTGGAGGATTACCT | TTCACCCTCTAGAAATGAAGC |
| *RPM1* | XP_107778129 | TGGAGATTGA GGTGCAACTC A | AGGTTCTGTGTATAGCTCCTTG |
| *RPS4* | XP_107768609 | T TAGCCTATTTCTTCCGGGGA | CGGCTGAACCATAACATTTCT |
| *PIK1* | XP_107759400 | CTTGACGACGGGTACCATCCA | TGGTCTTGCCCAAGCAACCAG |
