# Supplementary figures and images for: Broad-spectrum resistance mechanism of serine protease Sp1 in Bacillus licheniformis W10 via dual comparative transcriptome analysis
Source: Front Microbiol. 2022 Oct 4;13:974473. doi: 10.3389/fmicb.2022.974473 (PMC9577198; doi:10.3389/fmicb.2022.974473)

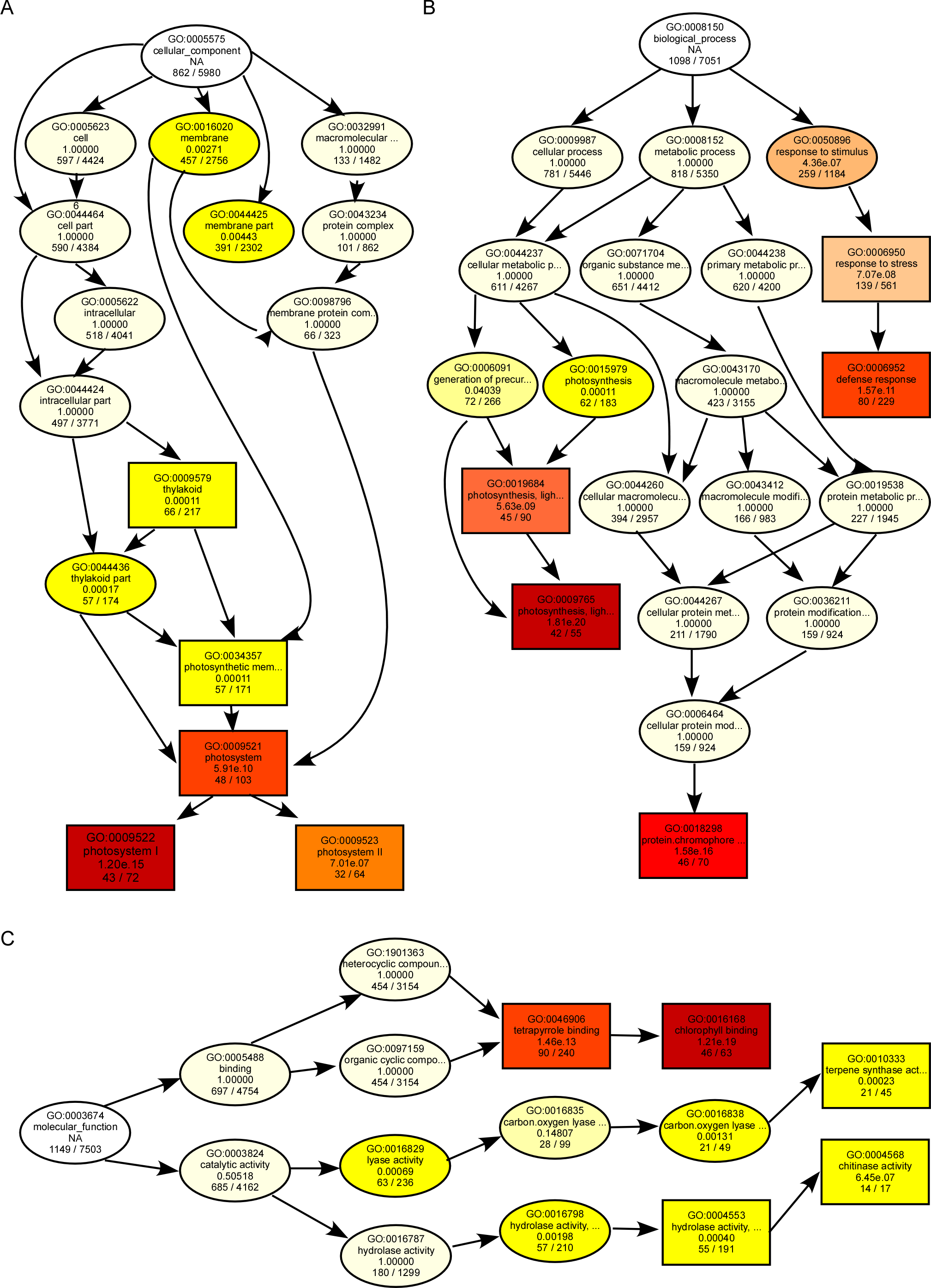

Supplement: Supplementary file 6 [file Image_6.tif]

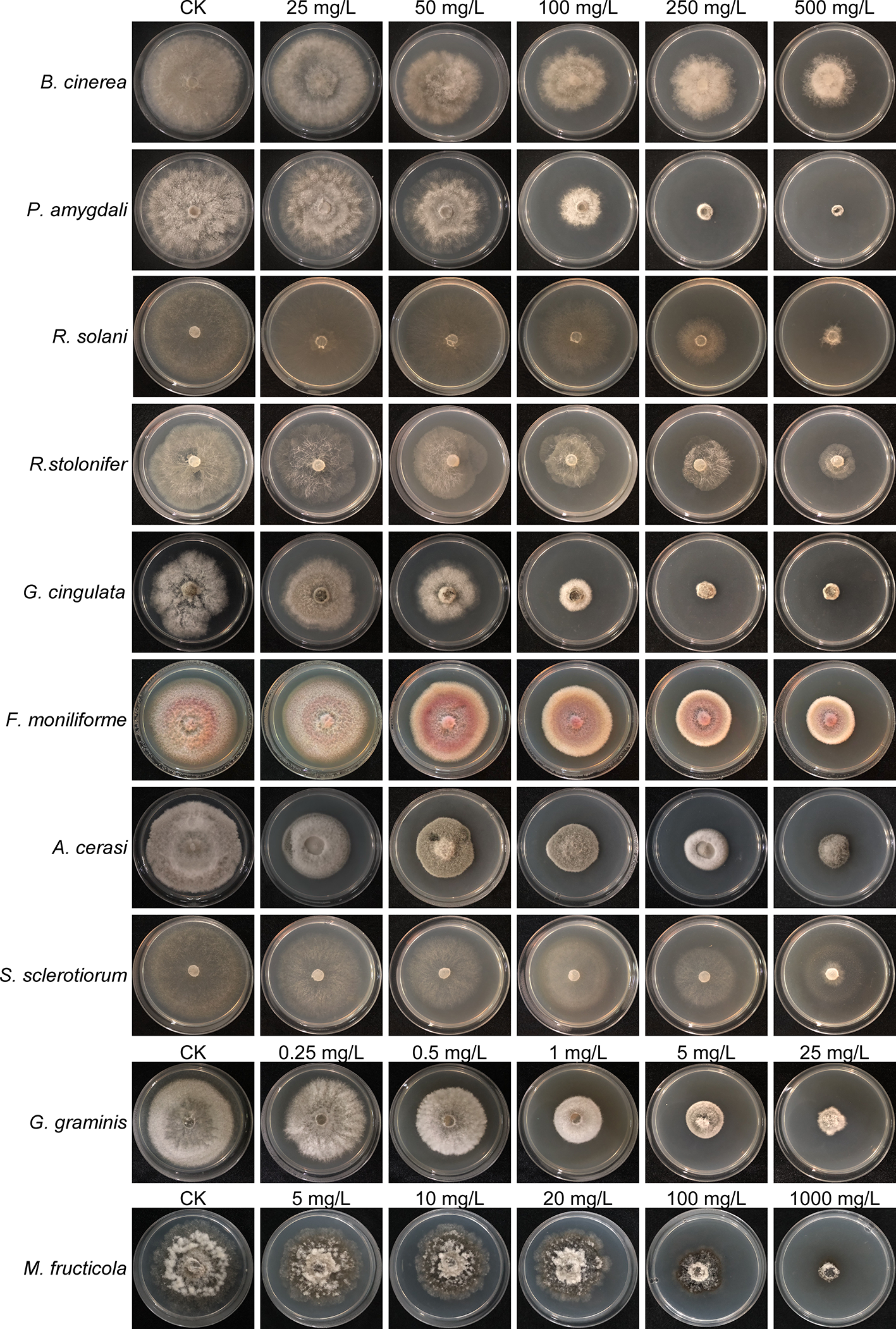

Supplement: Supplementary Figure S2 — Effects of different concentrations of W10-Sp1 protein to the mycelium of 10 kinds of plant pathogenic fungi. [file Image_2.tif]

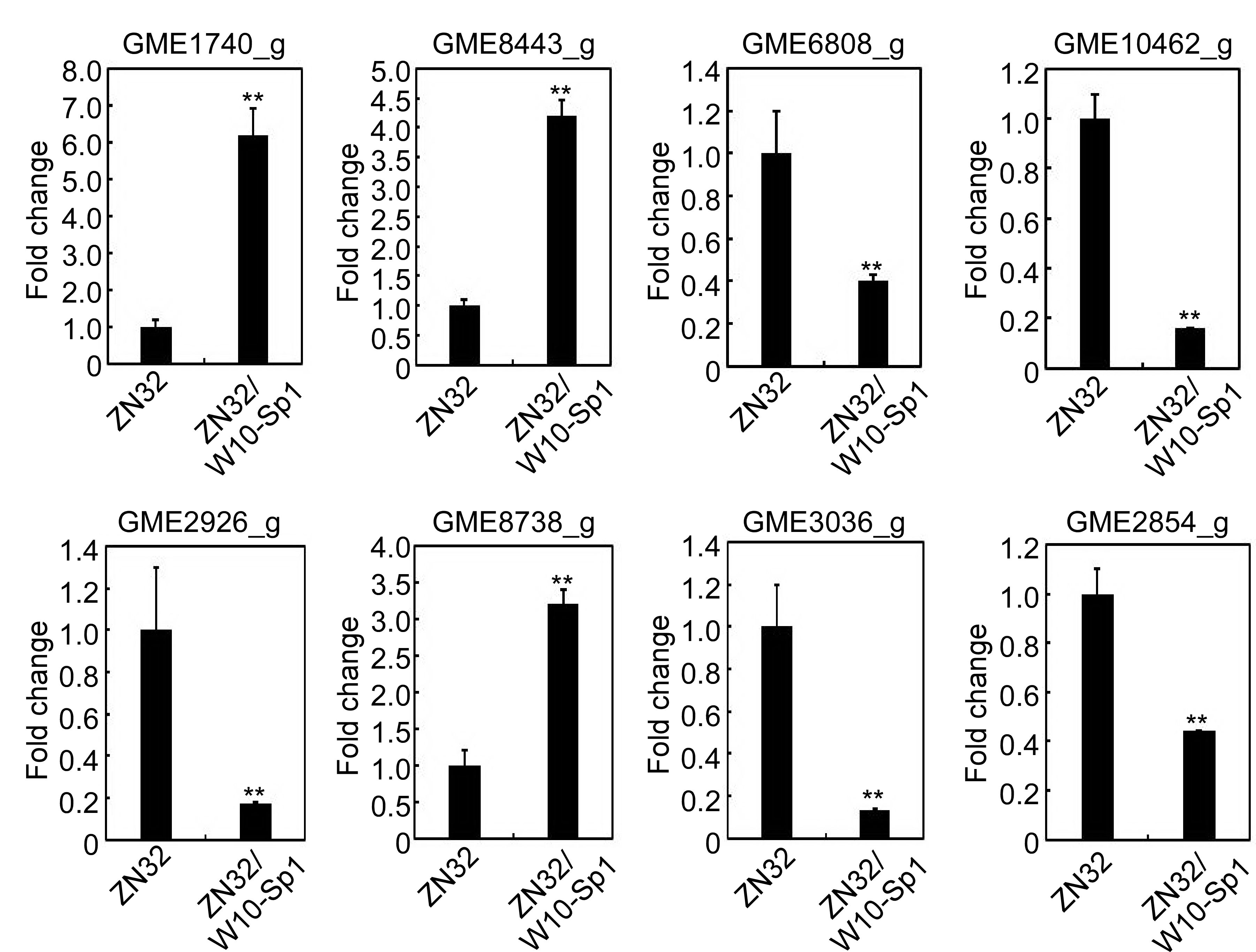

Supplement: Supplementary Figure S3 — Verification of RNA-Seq of PSp1-vs.- PCK by qRT-PCR. [file Image_3.tif]

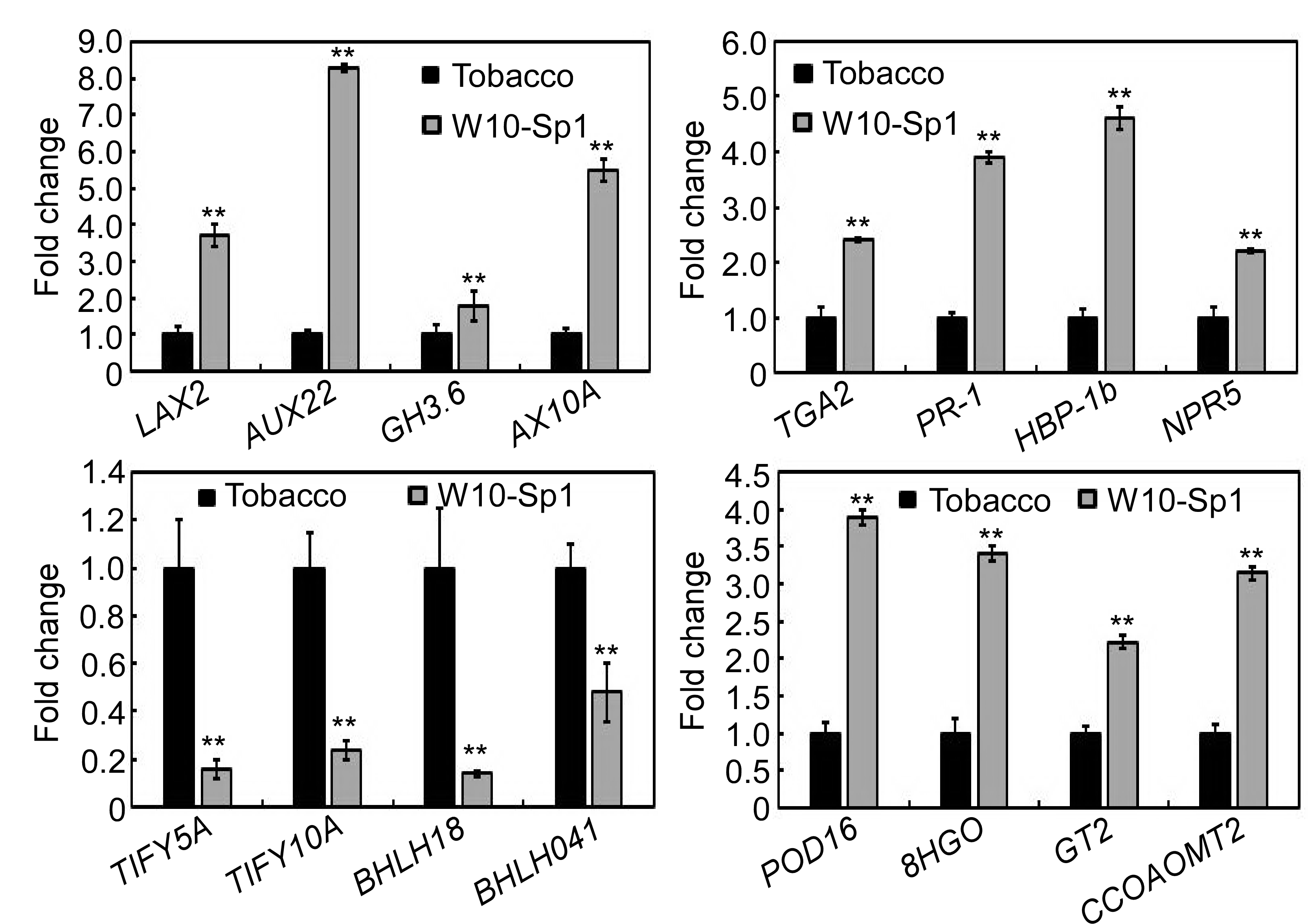

Supplement: Supplementary Figure S4 — Verification of RNA-Seq of TSp1-vs.- TCK by qRT-PCR. [file Image_4.tif]
